# Interaction of Glutaric Aciduria Type 1-Related glutaryl-CoA Dehydrogenase with Mitochondrial Matrix Proteins

**DOI:** 10.1371/journal.pone.0087715

**Published:** 2014-02-03

**Authors:** Jessica Schmiesing, Hartmut Schlüter, Kurt Ullrich, Thomas Braulke, Chris Mühlhausen

**Affiliations:** 1 Department of Biochemistry, Children's Hospital, University Medical Center Hamburg-Eppendorf, Hamburg, Germany; 2 Department of Clinical Chemistry, Laboratory for Mass Spectrometric Proteomics, University Medical Center Hamburg-Eppendorf, Hamburg, Germany; Universidade Nova de Lisboa, Portugal

## Abstract

Glutaric aciduria type 1 (GA1) is an inherited neurometabolic disorder caused by mutations in the *GCDH* gene encoding glutaryl-CoA dehydrogenase (GCDH), which forms homo- and heteromeric complexes in the mitochondrial matrix. GA1 patients are prone to the development of encephalopathic crises which lead to an irreversible disabling dystonic movement disorder. The clinical and biochemical manifestations of GA1 vary considerably and lack correlations to the genotype. Using an affinity chromatography approach we report here for the first time on the identification of mitochondrial proteins interacting directly with GCDH. Among others, dihydrolipoamide S-succinyltransferase (DLST) involved in the formation of glutaryl-CoA, and the β-subunit of the electron transfer flavoprotein (ETFB) serving as electron acceptor, were identified as GCDH binding partners. We have adapted the yellow fluorescent protein-based fragment complementation assay and visualized the oligomerization of GCDH as well as its direct interaction with DLST and ETFB in mitochondria of living cells. These data suggest that GCDH is a constituent of multimeric mitochondrial dehydrogenase complexes, and the characterization of their interrelated functions may provide new insights into the regulation of lysine oxidation and the pathophysiology of GA1.

## Introduction

The inherited neurodegenerative disorder glutaric aciduria type 1 (GA1, OMIM 231670) is caused by mutations in the gene for the mitochondrial matrix enzyme glutaryl-CoA dehydrogenase (GCDH, E.C. 1.3.99.7). GCDH belongs to the acyl-CoA dehydrogenase family of mitochondrial flavoproteins and catalyzes the oxidative decarboxylation of glutaryl-CoA in the degradative pathway of the amino acids lysine, hydroxylysine and tryptophan [1], [2]. The heterodimeric electron transfer flavoprotein (ETF) transfers electrons from GCDH to the respiratory chain [3], [4]. Mutations in the *GCDH* gene lead to formation and accumulation of the dicarboxylates glutaric acid (GA) and 3-hydroxyglutaric acid (3OHGA) in tissues and body fluids. Affected patients are at risk to develop encephalopathic crises triggered by catabolic situations such as infectious diseases, fever, vomiting or diarrhea. During crises a further increase of GA and 3OHGA concentrations were observed, accompanied by the selective destruction of striatal neurons with a subsequent development of an irreversible dystonic/dyskinetic movement disorder [4], [5]. Newborn screening programs allow the early identification of GA1 patients and the initiation of lysine and tryptophan restricted diet therapy prior to the development of encephalopathic crises [6]. Considerable variation in severity of the clinical and biochemical phenotype is observed showing no correlation to the genotype of the patients [7], [8]. More than 150 different mutations in the *GCDH* gene with predominance in specific populations have been described, which lead to a wide spectrum of clinical symptoms in GA1 patients ranging from an asymptomatic course to severe disabling dystonia [8]–[10].

The GCDH is synthesized as a precursor protein of 438 amino acids. After import into mitochondria the 44 N-terminal amino acid mitochondrial targeting sequence is cleaved off [9], and the assembly of four GCDH monomers containing a non-covalently bound flavin adenine dinucleotide (FAD) results in the enzymatically active tetrameric protein complex [11]. In addition to homotetramerization, cross-link experiments revealed that GCDH forms heteromeric higher molecular mass protein complexes with so far unidentified interaction partners [12].

In this report we used GCDH affinity chromatography, co-precipitation and protein complementation assays to identify and verify dihydrolipoamide S-succinyltransferase (DLST) and the electron transfer flavoprotein subunit beta (ETFB) as GCDH interacting proteins.

## Materials and Methods

### Antibodies

Rabbit anti-human GCDH antibody was kindly provided by Dr. S. I. Goodman (University of Colorado Health Sciences Center, Denver). The polyclonal mouse anti-human DLST and rabbit anti-human ETFA antibodies were purchased from Sigma (Munich, Germany), rabbit anti-human ETFB from Abcam (Cambridge, UK), and rabbit anti-LC3 from Abgent (San Diego, USA). The monoclonal mouse anti-GFP antibody was obtained from Roche (Mannheim, Germany) and rabbit anti-MnSOD from Millipore (Billerica, USA). Peroxidase-conjugated goat anti-rabbit IgG and goat anti-mouse IgG was from Dianova (Hamburg, Germany). HRP-conjugated anti-V5 antibody, monkey anti-mouse IgG coupled to Alexa Fluor 488 and goat anti-rabbit IgG coupled to Alexa Fluor 546 were from Invitrogen (Karlsruhe, Germany).

### DNA constructs

The human GCDH-Myc in the pcDNA6.2/V5/GW/TOPO vector has been described previously [12]. The LC3-GFP in the pEGFP-N1 (Clontech, Saint-Germain-en-Laye, France) vector was kindly provided by Dr. G. Galliciotti (this institute). The human *DLD* and *DLST* cDNAs (GenBank™ accession numbers NM_000108.3 and NM_001933.4, respectively) were isolated from total cDNA by PCR using Taq® polymerase (Amersham, Freiburg, Germany) and subcloned with a 3′ His_6_ sequence into pcDNA3.1/V5-His-TOPO using the corresponding TOPO TA Expression Kit (Invitrogen). The human *ETFA* and *ETFB* cDNA (GenBank™ accession numbers NM_000126.3 and NM_001985.2, respectively) were kindly provided by Dr. P. Bross (Aarhus University, Denmark) and isolated from the pCRII-ETFA and -ETFB vectors [13] by PCR using Phusion® polymerase (Thermo Scientific, St. Leon-Rot, Germany) and subcloned with a 3′ His_6_ sequence into the pcDNA3.1D/V5-His-TOPO vector using the corresponding Directional TOPO Expression Kit (Invitrogen). HMGCL cDNA was kindly provided by Dr. S. Gersting, (LMU Munich, Germany) and cloned into the pcDNA3.1D/V5-His-TOPO as described above. For expression in bacteria, the mature *GCDH, ETFA* and *ETFB* cDNA were additionally cloned into the pET28a(+) vector (Merck, Darmstadt, Germany) as 3′ or 5′ His_6_-fusion constructs, using NcoI and HindIII (*GCDH*) or NdeI and HindIII (*ETFA, ETFB*), respectively. For the protein complementation assay, DNA sequences of YFP fragments 1 (YFP1; amino acids 1–158) and 2 (YFP2; amino acids 159–239) with an introduced 5′-linker (GGGGS)_2_ were amplified by PCR using the vectors pcDNA3-MCFD2-cYFP1 and pcDNA3-MCFD2-cYFP2 (kindly provided by Dr. H.P. Hauri, University of Basel, Switzerland [14]) as template. Afterwards, the YFP1 and YFP2 cDNA was subcloned 3′ of DLST, HMGCL and GCDH into pcDNA3.1 by using the restriction enzymes EcoRV and XbaI. For the subcloning into pcDNA3.1-ETFA and -ETFB vector, EcoRV and XhoI were used. All expression vectors were sequenced (Seqlab, Göttingen, Germany). Primers used for the generation of all constructs are listed in Table S1.

### Cell culture and transfection

Baby hamster kidney 21 (BHK) cells and HeLa cells were cultured in Dulbecco's modified eagle medium (DMEM; Invitrogen) supplemented with 10% fetal calf serum (FCS; PAA Laboratories, Cölbe, Germany) and penicillin/streptomycin (Invitrogen). Cells grown on 10-cm plates were transfected with the indicated cDNAs using jetPEI™ Transfection Reagent (Peqlab Biotechnology, Erlangen, Germany) according to the manufacturer's instructions. The cells were used 24 h after transfection.

### Isolation of mitochondrial matrix proteins

Heavy mitochondrial fractions were prepared from pig liver (Ellegaard Göttinger Minipigs ApS, Dalmose, Denmark) using the basic protocol 1, previously described for rat liver [15], except centrifugation of the samples at 9,500 g. Purified mitochondria were resuspended in 0.25 M sucrose (40 mg/ml), followed by sequential fractionation into outer membranes, inner membranes and matrix proteins as described for rat liver [16].

### Preparation of GCDH affinity matrix

*GCDH* cDNA encoding the mature enzyme was subcloned into the *E. coli* expression vector pET28a(+) (Merck, Darmstadt, Germany) supplied with a C-terminal His_6_-tag. *E. coli* BL21(DE3) (Merck) were transformed using standard procedures and were grown in 400 ml Luria-Bertani (LB) medium supplemented with kanamycin (50 µg/ml) at 37°C in a shaking incubator until OD_600_ of 0.6 was reached. Cells were than induced for 4 h with 0.5 mM isopropyl β-D-thiogalactopyranoside (IPTG, Roth, Karlsruhe, Germany). Cells were resuspended in lysis buffer (50 mM NaH_2_PO_4_, pH 7.8 containing 500 mM NaCl, 10 mM imidazole, 2 mg/ml lysozyme, 20 µg/ml DNase, 1% Triton X-100 and protease inhibitor cocktail), sonicated and centrifuged for 15 min at 5,000 g at 4°C. To the supernatant 0.2 ml Ni-NTA agarose (Invitrogen) was added, incubated by rotating for 4 h at 4°C, washed and bound proteins were eluted sequentially with 50 mM NaH_2_PO_4_, pH 7.8 containing 500 mM NaCl and 150 mM or 250 mM imidazole. Purified GCDH (2 mg) was coupled to 2 ml bed volume of Affi-Gel 10 (Bio-Rad, Munich, Germany) according to the manufacturer's instructions.

### Identification of GCDH-binding proteins

Mitochondrial matrix protein extracts (1 mg) were applied to the GCDH-affinity matrix and incubated for 12 h at 4°C on a rotating wheel. Unbound material was discarded, and the column was washed with 10 vol. buffer A (50 mM Hepes, pH 7.5 containing 5 mM KCl and 120 mM NaCl). Bound proteins were eluted with 0.5 ml buffer A containing 1.5 M NaCl and protease inhibitor cocktail. The eluate was analyzed by liquid chromatography-tandem mass spectrometry (LC-MS/MS) as described previously [17].

### GCDH co-precipitation experiments

HeLa cells grown on 10 cm plates were transfected with *DLST-His_6_* cDNA alone or co-transfected with cDNA of *GCDH-myc* or *LC3-GFP*. Twenty-four hours after transfection cells were lysed in buffer B (10 mM Tris/HCl pH 7.5 containing 150 mM NaCl, 0.5 mM EDTA, 0.5% NP40, 1 mM PMSF and protease inhibitor cocktail), centrifuged for 10 min at 14,000 g, and 4°C and the supernatants were incubated with 0.1 ml Ni-NTA agarose for 4 h at 4°C on a rotating wheel to bind DLST-His_6_ protein complexes. Unspecific bound proteins were removed by a series of washes (3×0.5 ml buffer B, without NP40) and DLST-protein complexes were recovered by solubilization of beads in SDS-sample buffer and by SDS-PAGE followed by western blotting.

### Pull-down precipitation experiments

An overnight starter culture of pET28-*ETFA or ETFB* was established in LB-medium supplemented with kanamycin (50 µg/ml). Two ml of the culture was added to 200 ml of LB media supplemented with 50 µg/ml kanamycin. The culture was grown with shaking at 37°C to OD_600_ = 0.6. The expression of recombinant ETFA/ETFB was induced by addition of 0.5 mM IPTG for additional 4 h shaking at 37°C. Cells were pelleted at 4,000 *g* for 15 min. The pellet was resuspended in 10 ml cold buffer C (50 mM NaH_2_PO_4_ pH 7.8, containing 1% Triton X-100, 300 mM NaCl, 10 mM imidazole, 1 mg/ml lysozyme, 0.1 mg/ml DNase and protease inhibitor cocktail) and disrupted by ultrasonic treatment (6 times 20 sec). The resulting lysate was centrifuged at 10,000 *g* for 5 min at 4°C. The protein concentration of the supernatant containing the soluble ETFA or ETFB was determined by the Bio-Rad protein assay. 1.5 ml Ni-NTA agarose columns were equilibrated with buffer C, loaded with 4 mg/ml protein of the supernatant containing ETFA/ETFB and incubated by gently shaking for 4 h at 4°C. Afterwards the resin was divided into two columns of 0.75 ml each and incubated for additional 2 h with 0.3 mg of BHK cell extracts overexpressing GCDH or LC3, respectively. Unbound proteins were removed by a series of washes using buffer C (without Triton X-100, lysozyme and DNase) and protein complexes were recovered by solubilization of beads in SDS-sample buffer and by SDS-PAGE followed by western blotting.

### Other methods

Protein concentrations were determined with the Bio-Rad protein assay (Munich, Germany). Western blotting was performed as previously described [12] using anti-GCDH (1∶5,000), anti-ETFA (1∶1,000), anti-ETFB (1∶1,000), anti-DLST (1∶500), anti-GFP (1∶1,000) and anti-LC3 (1∶200) antibodies. For YFP fluorescence microscopy transfected BHK cells were grown on glass coverslips for 6 h and transfected with 2.5 µg pcDNA3.1 vectors (Invitrogen) containing the YFP1 or YFP2 fusion constructs. After 24 h the cells were fixed with 4% paraformaldehyde in 10 mM phosphate buffer saline, pH 7.4 (PBS). After washing and DAPI staining (Roth, Karlsruhe, Germany; 1∶1,000) the cells were embedded in Mowiol (Merck). Fluorescence was detected and images were obtained using a Leica DMIRE2 digital scanning confocal microscope with TCS NT software (Leica Microscopy Scientific Instruments Group, Wetzlar, Germany).

## Results

### GCDH affinity chromatography

**Figure 1:**
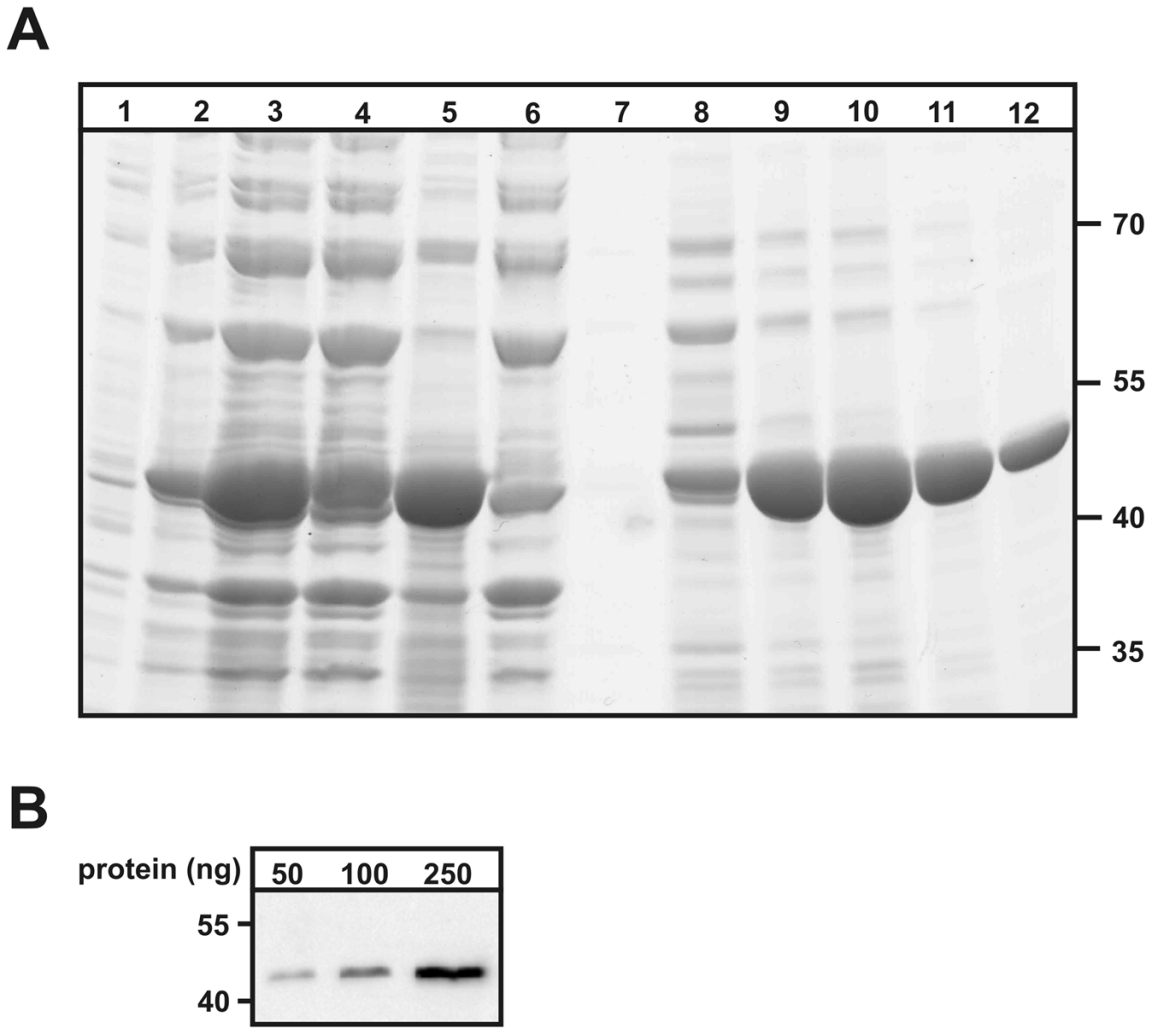
Ni-NTA purification of GCDH His_6_-fusion protein. (**A**) The expression of recombinant GCDH-His_6_ in *E. coli* was induced by the addition of IPTG (lane 1: before induction, lane 2: after induction). After 4 h *E. coli* cells were lysed by sonication (lane 3) and centrifuged (lane 4: pellet with insoluble proteins; lane 5: supernatant with soluble proteins). The supernatant with GCDH-His_6_ was incubated with Ni-NTA agarose and loaded on a column. Unbounded proteins (lane 6) were removed and the column was washed with increasing imidazole concentrations (lane 7: 10 mM imidazole; lane 8: 50 mM imidazole). Finally the GCDH-His_6_ protein was eluted in four steps with increasing imidazole concentrations (lane 9–11: 150 mM imidazole; lane 12: 250 mM imidazole). Samples were separated by SDS-PAGE (10% acrylamide) and proteins were visualized by Coomassie Blue staining. The positions of molecular mass marker proteins (in kDa) are indicated. (**B**) Validation of the purified GCDH-His_6_-fusion protein. Different amounts of purified GCDH-His_6_ protein were separated by SDS-PAGE (10% acrylamide) and analyzed by anti-GCDH western blotting. Representative pictures of n = 10 independent preparations are shown.

To identify proteins interacting with GCDH, we employed an affinity chromatography approach. Human GCDH fused to a His_6_-tag was expressed in *E. coli* and purified to homogeneity by Ni-chelate affinity chromatography (Fig. 1A). The purified GCDH has a molecular mass of 43 kDa in accordance with that predicted from the cDNA sequence. The identity of the purified polypeptide was confirmed by western blotting (Fig. 1B). The GCDH was immobilized covalently to beads and incubated with mitochondrial matrix protein extracts isolated from porcine liver (Fig. S1). Mass spectrometric analysis of polypeptides eluted with high salt buffer and digested with trypsin resulted in the identification of five mitochondrial matrix proteins, two inner mitochondrial membrane proteins and three peroxisomal proteins (Table 1; Table S2). Two mitochondrial matrix proteins, dihydrolipoamide S-succinyltransferase (DLST) and electron transfer flavoprotein subunit beta (ETFB) have been studied in more detail in this study because both proteins are directly involved in the degradation pathway of lysine and tryptophan. DLST is the E2 component of the 2-oxoglutarate dehydrogenase complex (OGDC), which catalyzes the oxidative decarboxylation of 2-oxoglutaric acid to glutaryl-CoA, the substrate of GCDH [2], [18]. ETF accepts electrons for subsequent transfer to ETF-ubiquinone oxidoreductase from nine acyl-CoA dehydrogenases, including GCDH [19].

**Table 1:** Mitochondrial proteins binding to GCDH.

Protein	Gene	Localization	Function
aldehyde dehydrogenase 2	ALDH2	M	oxidation of aldehydes to generate carboxylic acids
dihydrolipoamide-succinyltransferase	DLST	M	component of the 2-oxoglutarate dehydrogenase complex which catalyzes the conversion of 2-oxoglutarate to succinyl-CoA in the tricarboxylic acid cycle and the conversion of α-ketoadipate to glutaryl-CoA in the degradation pathway of lysine
electron transfer flavoprotein subunit beta	ETFB	M	ETF subunit acting as electron acceptor for several acyl-CoA dehydrogenases and transfer the electrons to the main mitochondrial respiratory chain via ETF-ubiquinone oxidoreductase
glutamate dehydrogenase 1	GLUD1	M	key enzyme in the nitrogen and glutamate/α-ketoglutarate metabolism
thioredoxin-dependent peroxide reductase	PRDX3	M	member of the peroxiredoxin family of antioxidant enzymes involved in cellular redox regulation
ATP synthase subunit alpha	ATP5A1	IM	part of the F_0_ domain of ATP synthase, functions as a proton channel
ATP synthase subunit beta	ATP5B	IM	part of the F_0_ domain of ATP synthase, functions as a proton channel

GCDH-His_6_ was immobilized on beads and incubated with isolated mitochondrial matrix proteins from pig liver. The identity of specifically co-purifying proteins was determined by LC-MS/MS.

M: mitochondrial matrix; IM: inner mitochondrial membrane.

**Figure 2:**
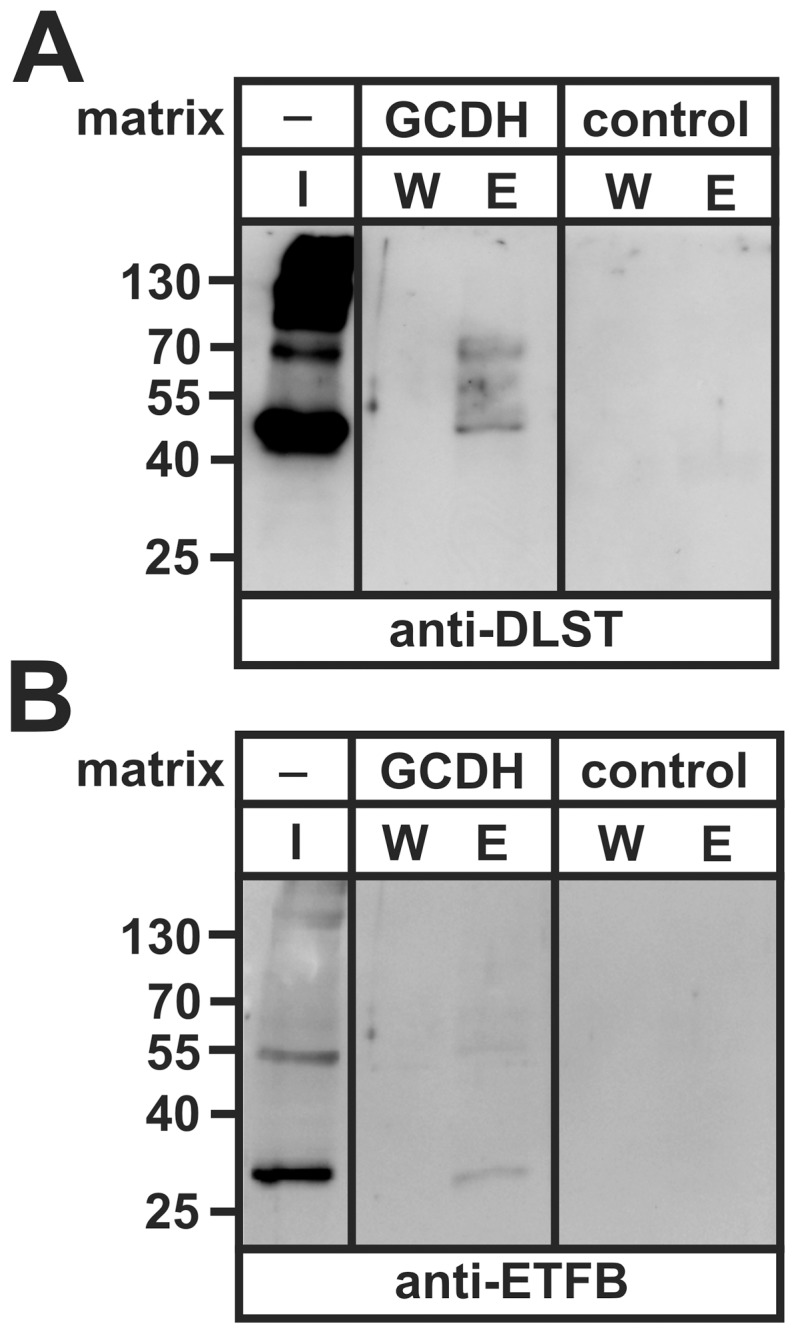
GCDH affinity chromatography of mitochondrial matrix extracts. Purified GCDH was covalently coupled to Affi-Gel 10 matrix and incubated with isolated mitochondrial matrix proteins. Aliquots of the loaded extract (input, I: 10% of total protein), the last wash fraction (W, 25%) and the high salt elution fraction (E, 100%) were separated by SDS-PAGE (10% acrylamide) and tested by anti-DLST (**A**) and anti-ETFB (**B**) western blotting. Non-coupled Affi-gel 10 beads were used as a control for unspecific binding. The positions of the molecular mass marker proteins (in kDa) are given. A representative blot of n = 3 independent preparations is shown.

DLST positive polypeptides of 69, 55 and 49 kDa have been detected in the elution fraction after GCDH affinity chromatography by western blotting (Fig. 2A). The 49 kDa DLST polypeptide represents most likely the E2 component of OGDC, whereas the 69 and 55 kDa polypeptides may represent E2 components of the pyruvate dehydrogenase complex (PDC) and branched-chain alpha-ketoacid dehydrogenase complex (BCKDC; [20]), which are highly homologous to DLST and may cross-react with the used DLST antibody. To exclude that binding of DLST to GCDH is mediated indirectly via the common E3 subunit dihydrolipoamide dehydrogenase (DLD) of OGDC, PDC and BCKDC, rather than a direct interaction, DLD-His_6_ co-precipitation experiments were performed. These experiments showed only a very weak GCDH-positive polypeptide, suggesting that DLD binds to GCDH indirectly via the directly bound DLST (Figure S2). The affinity elution fraction also contained the 28 kDa ETFB (Fig. 2B). Neither DLST nor ETFB bound to the control chromatography matrix.

### GCDH binds directly to DLST and ETFB

**Figure 3:**
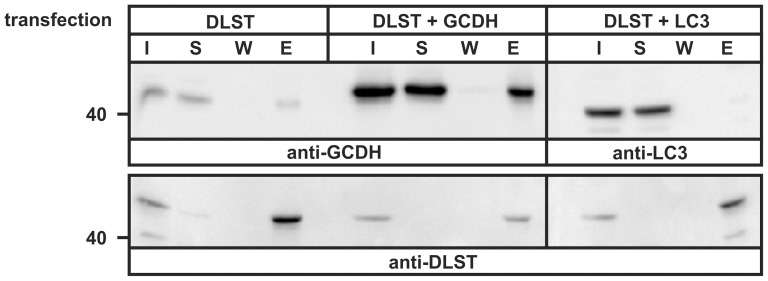
Co-Precipitation of DLST with GCDH. Extracts from HeLa cells overexpressing the DLST-His_6_ alone (DLST) or together with GCDH-Myc (DLST+GCDH) were incubated with Ni-NTA agarose for 4 h. Aliquots of the cell extract (input, I: 10% of total), the unbound protein supernatant after precipitation of Ni-NTA beads (S, 10%), the last wash (W, 25%) and the eluted fraction (E, 100%) representing bound proteins, were analyzed by successively exposing the blot to anti-GCDH and, after stripping, to anti-DLST antibodies. Extracts of HeLa cells overexpressing DLST-His_6_ and LC3-GFP (DLST+LC3) were used as negative control and analyzed by anti-LC3 western blotting. The expression of DLST was analyzed by anti-DLST western blotting. The position of the 40 kDa molecular mass marker protein is indicated. The figure shows a representative blot of n = 3 independent experiments.

To confirm the results of GCDH affinity chromatography, the interaction between GCDH and DLST was examined by co-precipitation experiments. When HeLa cells were transfected with cDNA encoding the DLST-His_6_ protein followed by Ni-NTA-agarose precipitation, about 2% of endogenous GCDH could be recovered from the beads (Fig. 3, DLST). After co-expression of DLST-His_6_ with GCDH-Myc (DLST+GCDH) a strong increase of GCDH in the eluted (E) fraction was observed. Non-bound GCDH was found in the supernatant (S). Co-expression of DLST with a cytosolic protein LC3-GFP (DLST+LC3) was used as a negative control. As expected, LC3-GFP did not bind to DLST-His_6_. Immunoreactive 40 kDa LC3-GFP polypeptides were only detectable in the input (I) and in the supernatant fraction (Fig. 3). The expression of the 49 kDa DLST-His_6_ protein used for precipitation was verified by anti-DLST immunoblotting.

**Figure 4:**
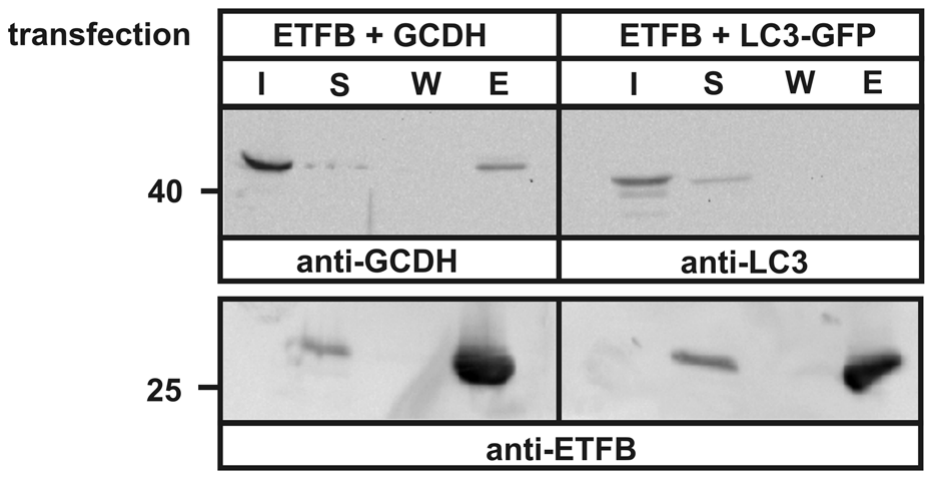
Binding of purified ETFB to GCDH. ETFB-His_6_ expressed and purified from *E. coli* was immobilized on Ni-NTA agarose and incubated with extracts from BHK cells overexpressing GCDH-Myc (ETFB+GCDH) for 2 h. Cell extracts overexpressing LC3-GFP were used as negative control (ETFB+LC3). Aliquots of cell extract (input, I: 10% of total), the unbound protein supernatant after precipitation of ETFB-Ni-NTA beads (S: 10%), last wash (W, 25%) and the eluted fraction (E, 100%), containing the bound proteins were separated by SDS-PAGE (10% acrylamide) and analyzed by anti-GCDH and anti-LC3 immunoblotting. The expression of ETFB used for pull-down was analyzed by anti-ETFB western blotting. The image shows representative blots of n = 5 independent experiments.

In order to confirm the interaction between GCDH and ETFB, we carried out His_6_-pulldown experiments. Purified ETFB-His_6_ immobilized on Ni-NTA agarose was incubated with cell extracts overexpressing GCDH-Myc. GCDH was recovered in the elution fraction from ETFB-agarose matrix (Fig. 4). No interaction was observed between ETFB-His_6_ and the cytosolic protein LC3-GFP. The expression of the 28 kDa ETFB-His_6_ was verified by anti-ETFB immunoblotting.

Since ETFB is known to form stable complexes with ETFA [21], we performed ETFA-His_6_ pulldown experiments. Very low amounts of GCDH-immunoreactive material were detected in the elution fraction, suggesting a weak binding of GCDH to ETFA (Fig. S3).

### YFP-based protein complementation assay (PCA)

**Figure 5:**
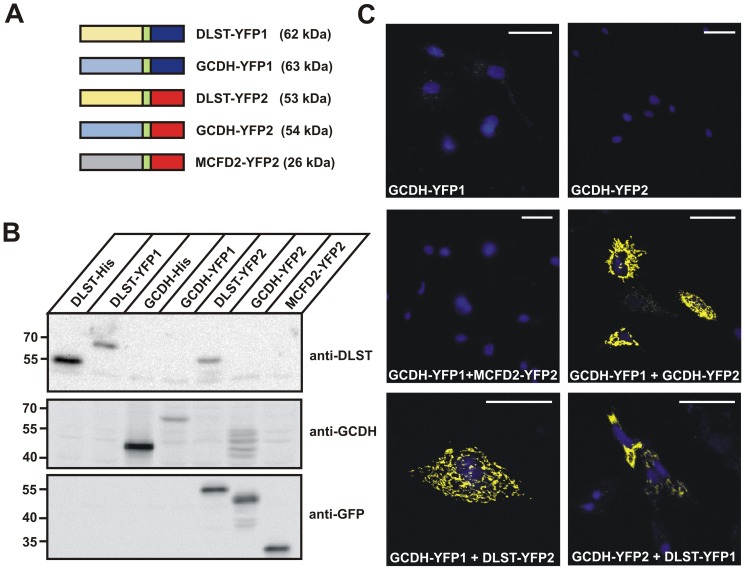
YFP fragment complementation assay demonstrates the interaction of GCDH with DLST *in vivo*. (**A**) Schematic composition of C-terminal YFP1 (dark blue) and YFP2 (red) fusion proteins of DLST, GCDH and MCFD2 used in this study. The 10-amino acid linker (GGGGS)_2_ is indicated in green. The calculated molecular masses of the fusion proteins are shown in brackets. The ERGIC marker protein MCFD2-YFP2 was used as negative control. (**B**) Expression analysis in BHK cells of all fusion proteins visualized by western blotting, using anti-DLST, anti-GCDH and anti-GFP antibodies. (**C**) Fluorescence microscopy of the indicated single or co-expressed fusion proteins. Strong YFP fluorescence was observed in cells co-expressing either GCDH-YFP1 with DLST-YFP2 or GCDH-YFP2 with DLST-YFP1. Nuclei were visualized using DAPI (blue). Scale bars = 40 µM. Representative images of n = 3 independent transfection experiments are shown.

**Figure 6:**
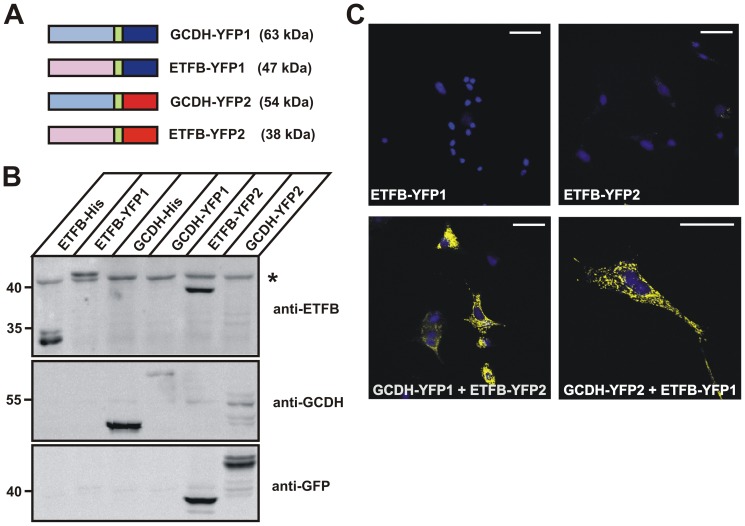
Interaction of GCDH with ETFB *in vivo*. (**A**) Schematic representation of GCDH and ETFB fusion proteins with YFP1 (dark blue) and YFP2 (red). The 10-amino acid linker (GGGGS)_2_ is indicated in green. The calculated molecular masses of the respective fusions proteins are shown in brackets. (**B**) The expression of the various fusion proteins used in this study were analyzed by western blotting. The 43 kDa band (*) reactive with the anti-ETFB antibody is unspecific. (**C**) Fluorescence microscopy of fixed BHK cells co-expressing GCDH and ETFB fusion proteins showed a strong YFP fluorescence. Nuclei were visualized using DAPI (blue). Scale bars = 40 µM. Representative images of n = 3 independent transfection experiments are shown.

To visualize and verify the interaction between GCDH and DLST as well as GCDH and ETFB in living cells, we adapted the protein fragment complementation assay (PCA) to the mitochondrial compartment. The assay relies on yellow fluorescent protein (YFP) fragments, YFP1 and YFP2, fused to two interacting proteins. Interaction of the fusion proteins brings the two YFP-fragments into close proximity allowing their folding into the active YFP structure [14], [22]. In this study GCDH, DLST, ETFB and ETFA were C-terminally either tagged with YFP1 (amino acids 1–158) or YFP2 (amino acids from 159 to 239) (Fig. 5A and 6A). As negative controls, we included the multiple coagulation factor deficiency protein 2 (MCFD2), localized in the endoplasmic reticulum-Golgi intermediate compartment (ERGIC) [23], and the mitochondrial matrix enzyme 3-hydroxy-3-methylglutaryl-CoA lyase (HMGCL) [24]. Individual expression of different YFP1- and YFP2-fusion proteins in BHK cells was confirmed by western blotting. Anti-GFP antibodies recognize YFP2-fusion proteins but not YFP1 constructs (Fig. 5B and 6B). To test the application of YFP-PCA for mitochondrial matrix proteins, we first studied the homooligomerization of GCDH. Strong YFP fluorescence was observed when GCDH-YFP1 and GCDH-YFP2 were co-expressed, indicating fragment complementation upon GCDH oligomerization (Fig. 5C). No YFP fluorescence was detected in BHK cells expressing GCDH-YFP1 or GCDH-YFP2 alone, demonstrating that YFP fragments *per se* have no intrinsic fluorescence (Fig. 5C). Co-expression of GCDH-YFP1 with DLST-YFP2 and GCDH-YFP2 with DLST-YFP1, respectively, resulted in a strong fluorescence signal (Fig. 5C).

Additionally, co-expression of GCDH-YFP1 and ETFB-YFP2 or GCDH-YFP2 and ETFB-YFP1 in BHK cells revealed a strong YFP fluorescence (Fig. 6C). No intrinsic YFP fluorescence was detected in BHK cells expressing ETFB-YFP1 or ETFB-YFP2 alone.

The GCDH-DLST and GCDH-ETFB fluorescence patterns are typical for mitochondria. Overlay of PCA-positive signals generated by co-expression of GCDH- with either DLST- or ETFB-YFP constructs with the counterstaining of the endogenous mitochondrial matrix protein manganese-dependent superoxide dismutase (MnSOD; Fig. S4) confirmed the localization of the interactions between GCDH and either DLST or ETFB in the mitochondrial matrix. In contrast to the strong signal intensity detecting the interaction between GCDH and ETFB distributed over the cell, the co-expression of GCDH-YFP1 and ETFA-YFP2 showed a dramatically restricted number of stained mitochondria (Fig. S5). As expected, the co-expression of ETFA-YFP2 with ETFB-YFP1 revealed a strong PCA signal, confirming that the constructs are functional.

No YFP fluorescence has been observed when GCDH-YFP1 was co-expressed with the ERGIC marker protein MCFD2 fused to YFP2, or with the YFP2-fused mitochondrial matrix protein HMGCL, verifying the specific interaction between GCDH and DLST or ETFB (Fig. 5C; Fig. S4). The data demonstrate the selectivity and specificity of GCDH-DLST and GCDH-ETFB interactions in the mitochondrial compartment.

Both co-precipitation and YFP-PCA verified the direct interactions between GCDH and DLST as well as GCDH and ETF, in particular ETFB.

## Discussion

In the present study we have identified protein-protein interactions between GCDH, a mitochondrial matrix enzyme involved in the degradation of lysine and tryptophan, and several other mitochondrial metabolic proteins using immobilized GCDH affinity chromatography coupled with mass spectrometric proteome analysis. Among these proteins, dihydrolipoamide S-succinyltransferase (DLST) and electron transfer flavoprotein subunit beta (ETFB) were examined in more detail.

DLST constitutes the oligomeric E2-core subunit of the large mitochondrial matrix 2-oxoglutarate dehydrogenase complex (OGDC) containing additionally multiple copies of 2-oxoglutarate dehydrogenase (E1) and dihydrolipoamide dehydrogenase (E3). OGDC catalyzes the rate-limiting step of oxidative decarboxylation of both α-ketoglutarate and α-ketoadipate to succinyl- and glutaryl-CoA, respectively, in the tricarboxylic acid (TCA) cycle [25], [26]. In addition to OGDC, pyruvate dehydrogenase (PDC) and branched-chain α-ketoacid dehydrogenase complex (BCKDC) belong to the family of α-ketoacid dehydrogenase multienzyme complexes containing homologous DLST-E2 subunits and may explain the 55 and 69 kDa DLST immunoreactive bands detected in the elution fraction. The enzymatic activity of OGDC is regulated by feedback inhibition of the glutaryl-CoA reaction product which has been shown to inhibit the DLST subunit of OGDC *in vitro* [18]. The interaction between GCDH and DLST suggests that both consecutive enzymes function in a multienzyme complex to allow sufficiently short distance for efficient oxidative decarboxylation of glutaryl-CoA to crotonyl-CoA.

The other GCDH binding partner, ETFB, forms with ETFA an FAD-containing heterodimer that serves as electron acceptor for at least nine mitochondrial matrix flavoprotein dehydrogenases of fatty acid oxidation and amino acid catabolism. Subsequently the electrons are passed through the membrane-bound ETF ubiquinone oxidoreductase to the respiratory chain [21]. Defects in ETF cause multiple acyl-CoA dehydrogenase (MAD) deficiency, also called glutaric aciduria type 2 (GA2) [27].

In addition, three other mitochondrial matrix proteins have been identified as potential GCDH-binding partners. First, the aldehyde dehydrogenase 2 (ALDH2) is an allosteric tetrameric enzyme that catalyzes the oxidation of ethanol-derived acetaldehyde to acetate [28]. Second, peroxiredoxin 3 (PRDX3) is a peroxidase exclusively localized in the mitochondrial matrix and has protective effects to mitochondrial oxidative stress [29]. Of note, PRDX3 has been reported to be closely linked to the DLST expression in murine adipocytes [30]. Third, glutamate dehydrogenase (GLUD1) is a homohexameric enzyme that catalyzes the reversible oxidative deamination of glutamate to α-ketoglutarate and plays a central role in the nitrogen and glutamate metabolism as well as the cellular energy homeostasis. In mammals, GLUD1 is highly regulated by allosteric effectors [31]. Dominant mutations in GLUD1 that cause a loss of allosteric inhibition lead to unusual hyperinsulinism/hyperammonemia syndrome [32]. Interestingly, ammonium accumulation and occasionally hypoglycemia have been reported in a rat 3D brain cell model of GA1 and in GA1 children, respectively [33], [34], supporting a potential functional interaction between Glud1 and GCDH. Furthermore, in the brain Glud1 is predominantly expressed in astrocytes, and the loss of Glud1 reduces the oxidative catabolism of glutarate to α-ketoglutarate [35], which secondarily impairs the anaplerotic transfer of TCA cycle intermediates to neurons [36]. Since the accumulation of GA and 3OHGA associated with GCDH-deficiency also inhibits the astrocytic efflux and neuronal uptake of TCA cycle intermediates [37], it is tempting to speculate that patients with GCDH mutations interfering with the binding or allosteric control of Glud1 activity are more severely affected than others. However, the potential interactions of GCDH with ALDH2, PRDX3, and Glud1 detected by affinity chromatography have to be confirmed by detailed experimental studies as done for ETFB and DLST. Furthermore, studies are needed to examine the biological significance of the interaction of the various mitochondrial matrix and inner mitochondrial membrane proteins with GCDH, which might be important for modulation of GCDH activity or the coordinated allosteric control of other multimeric dehydrogenase complexes.

At present the amino acid residues on GCDH, involved in the direct binding to DLST and ETFB are unknown. These residues are predicted to be located at the surface of GCDH. About 20 mutations found in GA1 patients affect amino acid residues on the GCDH surface [8]–[10] associated with residual GCDH activities of 0–30% of controls and a wide spectrum of clinical symptoms in the respective GA1 patients. Thus, the p.Met263Val mutation, exhibiting 30% residual enzyme activity in patient fibroblasts [38], is located at the surface of GCDH, and failed to form heterologous GCDH-protein complexes upon chemical cross-linkage [12]. Co-crystalization of ETF with another mitochondrial dehydrogenase, medium-chain acyl-CoA dehydrogenase (MCAD), revealed that ETFB interacts with nine residues in the N-terminal domain of MCAD, Glu22, Phe 23, Thr26, Glu34, Gly60, Thr64, Leu73, Leu75 and Ile83, which mediate hydrogen bonds to ETFB or form a hydrophobic pocket [39]. Because MCAD and GCDH share a sequence homology of 28%, it appeared likely that also amino acid residues in the N-terminal domain of GCDH are involved in ETFB binding. A direct comparison, however, of the amino acids of the GCDH protein that correspond to the ETFB-binding residues in the MCAD protein, revealed a low homology with only 2 out of 9 identical amino acids between GCDH and MCAD, Thr26 and Gly60 (Fig. S6). Further mutational analyses on GCDH are needed to identify the residues involved in ETFB binding.

Taken together, in this study five mitochondrial matrix proteins have been identified to be able to bind to GCDH. Among these, the physical interaction between DLST, constituting an oligomeric core subunit of the multienzyme α-ketoglutarate dehydrogenase complex, and ETFB, serving as electron acceptor for several mitochondrial dehydrogenases, and GCDH have been verified with different experimental approaches. The identification of the first GCDH interacting proteins provides new insights into the functional linkage between multienzyme complexes required for efficient metabolism of glutaryl-CoA, and its role in the pathogenesis of glutaric aciduria type 1.

## Supporting Information

Figure S1 **Isolation of mitochondrial matrix proteins.** Crude mitochondrial extracts were fractionated into outer membrane, inner membrane and matrix proteins. Ten µg of the fraction with mitochondrial matrix proteins was separated by SDS-PAGE (10% acrylamide) and proteins were visualized by Coomassie Blue staining. The positions of molecular mass marker proteins (in kDa) are indicated.
(JPG)

Figure S2 **Co-precipitation of DLD with GCDH.** Extracts from HeLa cells overexpressing DLD-His_6_-V5 together with GCDH-YFP2 (DLD+GCDH) or together with LC3-GFP (DLD+LC3) were incubated with Ni-NTA agarose for 4 h. Aliquots of the cell extract (input, I: 10% of total), the unbound protein supernatant after precipitation of Ni-NTA beads (S, 10%), and the eluted fraction (E, 100%) representing bound proteins, were analyzed by anti-GFP western blotting detecting GCDH-YFP2 and LC3-GFP. Extracts of HeLa cells overexpressing DLD-His_6_-V5 and LC3-GFP (DLD+LC3) were used as negative control. The expression of DLD was analyzed by anti-V5 western blotting. The positions of the 55 and 40 kDa molecular mass marker proteins are indicated. Arrow: DLD-immunoreactive 52 kDa-band.
(JPG)

Figure S3 **Binding of purified ETFA to GCDH.** ETFA-His_6_ expressed and purified from *E. coli* was immobilized on Ni-NTA agarose and incubated with extracts from BHK cells overexpressing GCDH-Myc (ETFA+GCDH) for 2 h. Cell extracts overexpressing LC3-GFP were used as negative control (ETFA+LC3). Aliquots of cell extract (input, I: 10% of total), the unbound protein supernatant after precipitation of ETFA-Ni-NTA beads (S: 10%), last wash (W, 25%) and the eluted fraction (E, 100%), containing the bound proteins were separated by SDS-PAGE (10% acrylamide) and analyzed by anti-GCDH and anti-LC3 immunoblotting. The expression of ETFA used for the pull-down experiments was analyzed by anti-ETFA western blotting. The image shows representative blots of n = 5 independent experiments.
(JPG)

Figure S4 **YFP fragment complementation assay and mitochondrial counterstaining.** (**A**) Schematic composition of C-terminal YFP1 (dark blue) and YFP2 (red) fusion proteins of GCDH, ETFB, DLST, and HMGCL used in this study. The 10-amino acid linker (GGGGS)_2_ is indicated in green. The calculated molecular masses of the fusion proteins are shown in brackets. The mitochondrial matrix protein HMGCL was used as negative control. (**B**) Expression analysis in HeLa cells of all fusion proteins visualized by western blotting, using anti-GCDH and anti-GFP antibodies. *endogenous GCDH protein. (**C**) Fluorescence microscopy of the indicated co-expressed fusion proteins. Strong YFP fluorescence was observed in cells co-expressing GCDH-YFP1 with either GCDH-YFP2, ETFB-YFP2, or DLST-YFP2. No YFP fluorescence signal was observed when GCDH-YFP1 was co-expressed with HMGCL-YFP2. Nuclei were visualized using DAPI (blue). Mitochondria were counterstained with anti-MnSOD antibody. Merged signals indicate co-localization of PCA signal with MnSOD-positive mitochondria. Scale bars = 40 µm (merge) or 10 µm (zoom).
(JPG)

Figure S5 **Interaction of GCDH with ETFA *in vivo*.** (**A**) Fluorescence microscopy of fixed BHK cells co-expressing GCDH-YFP1 and ETFA-YFP2 fusion proteins showed a YFP fluorescence signal only in few mitochondria. (**B**) In contrast, co-expression of ETFB-YFP1 with ETFA-YFP2 revealed a strong fluorescence signal with a typical mitochondrial expression pattern. Nuclei were visualized using DAPI (blue). Scale bars = 40 µm.
(JPG)

Figure S6 **Comparison of the ETFB-binding site of MCAD with GCDH.** Sequence alignment of mature GCDH and MCAD proteins. Identical amino acids are presented in blue. MCAD amino acid residues that have been reported to interact with ETFB [39] and to form hydrogen bonds (yellow), a hydrophobic pocket (green), or both (orange), are indicated.
(JPG)

Table S1 **Sequences of primers used in this study.**
(DOC)

Table S2 **LC-MS/MS analyses of the GCDH affinity chromatography elution fraction identifying mitochondrial and non-mitochondrial proteins.**
(DOC)
