# Supplementary figures and images for: Interaction of Glutaric Aciduria Type 1-Related glutaryl-CoA Dehydrogenase with Mitochondrial Matrix Proteins
Source: PLoS One. 2014 Feb 3;9(2):e87715. doi: 10.1371/journal.pone.0087715 (PMC3912011; doi:10.1371/journal.pone.0087715)

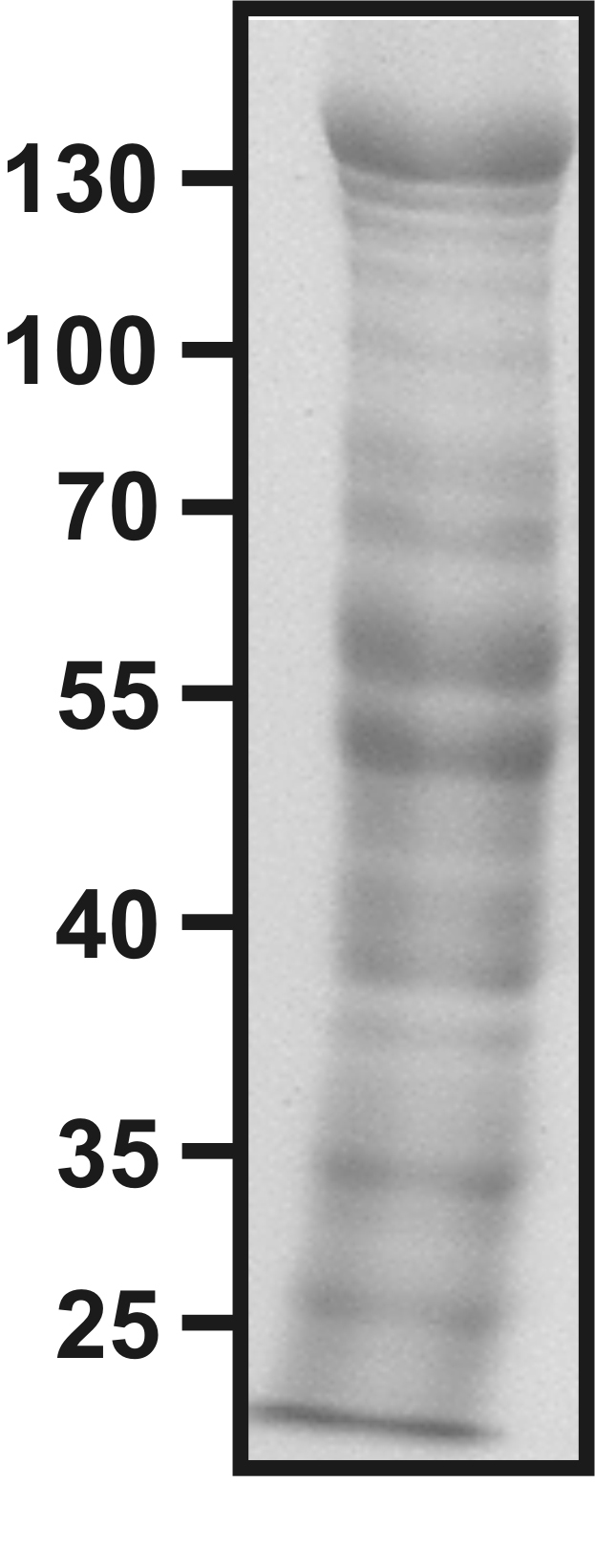

Supplement: Figure S1 — Isolation of mitochondrial matrix proteins. Crude mitochondrial extracts were fractionated into outer membrane, inner membrane and matrix proteins. Ten µg of the fraction with mitochondrial matrix proteins was separated by SDS-PAGE (10% acrylamide) and proteins were visualized by Coomassie Blue staining. The positions of molecular mass marker proteins (in kDa) are indicated. (JPG) [file pone.0087715.s001.jpg]

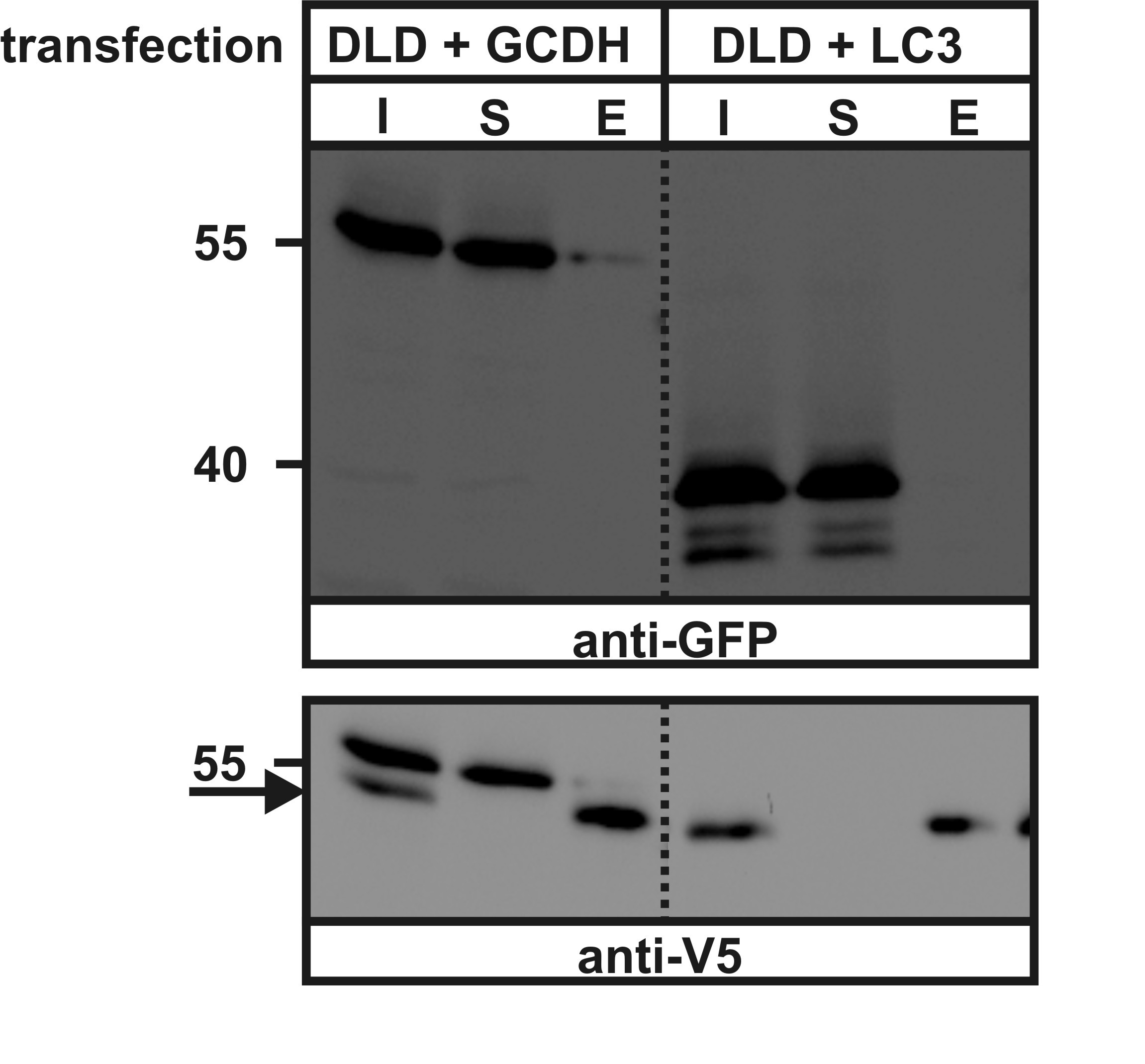

Supplement: Figure S2 — Co-precipitation of DLD with GCDH. Extracts from HeLa cells overexpressing DLD-His6-V5 together with GCDH-YFP2 (DLD+GCDH) or together with LC3-GFP (DLD+LC3) were incubated with Ni-NTA agarose for 4 h. Aliquots of the cell extract (input, I: 10% of total), the unbound protein supernatant after precipitation of Ni-NTA beads (S, 10%), and the eluted fraction (E, 100%) representing bound proteins, were analyzed by anti-GFP western blotting detecting GCDH-YFP2 and LC3-GFP. Extracts of HeLa cells overexpressing DLD-His6-V5 and LC3-GFP (DLD+LC3) were used as negative control. The expression of DLD was analyzed by anti-V5 western blotting. The positions of the 55 and 40 kDa molecular mass marker proteins are indicated. Arrow: DLD-immunoreactive 52 kDa-band. (JPG) [file pone.0087715.s002.jpg]

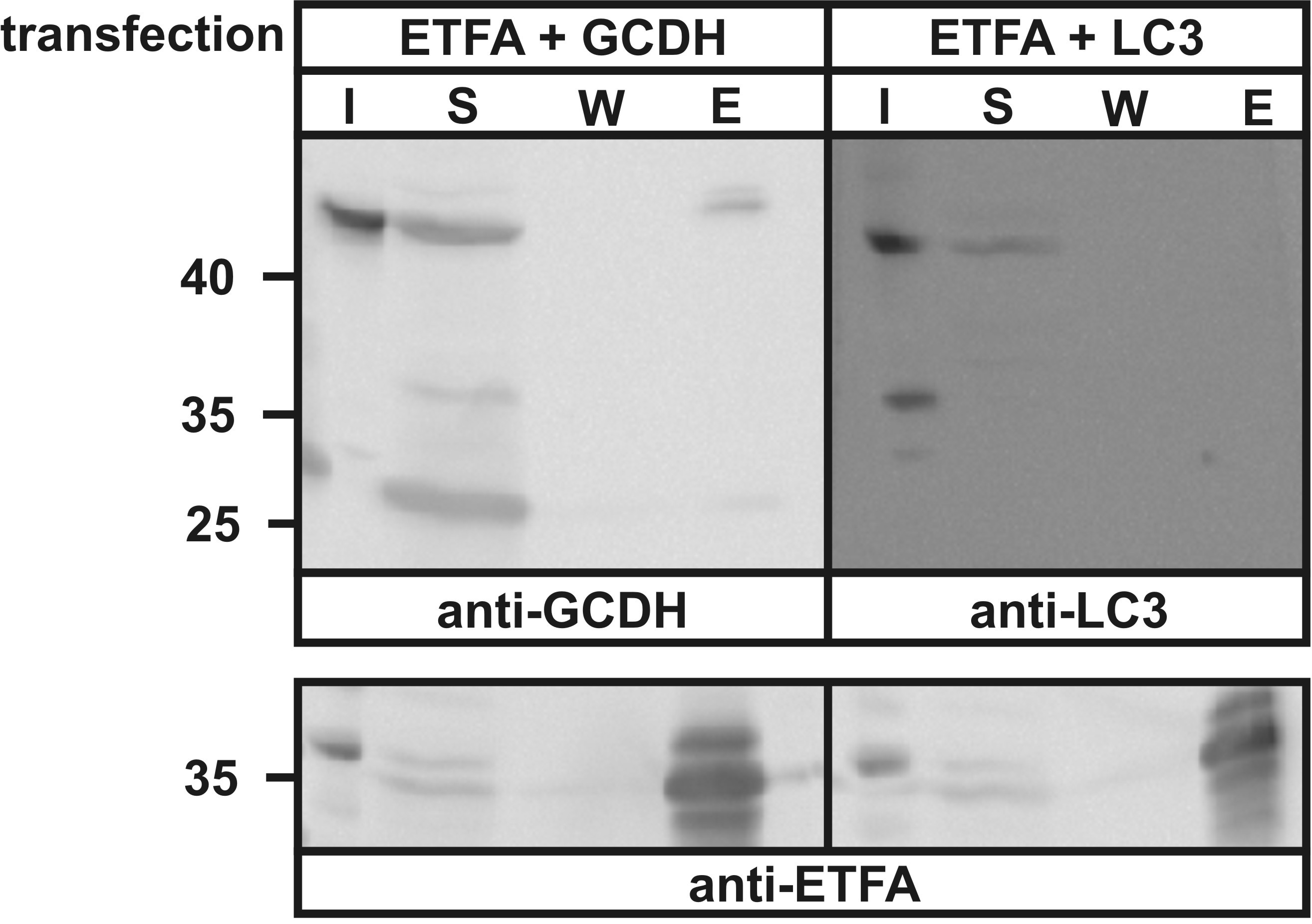

Supplement: Figure S3 — Binding of purified ETFA to GCDH. ETFA-His6 expressed and purified from E. coli was immobilized on Ni-NTA agarose and incubated with extracts from BHK cells overexpressing GCDH-Myc (ETFA+GCDH) for 2 h. Cell extracts overexpressing LC3-GFP were used as negative control (ETFA+LC3). Aliquots of cell extract (input, I: 10% of total), the unbound protein supernatant after precipitation of ETFA-Ni-NTA beads (S: 10%), last wash (W, 25%) and the eluted fraction (E, 100%), containing the bound proteins were separated by SDS-PAGE (10% acrylamide) and analyzed by anti-GCDH and anti-LC3 immunoblotting. The expression of ETFA used for the pull-down experiments was analyzed by anti-ETFA western blotting. The image shows representative blots of n = 5 independent experiments. (JPG) [file pone.0087715.s003.jpg]

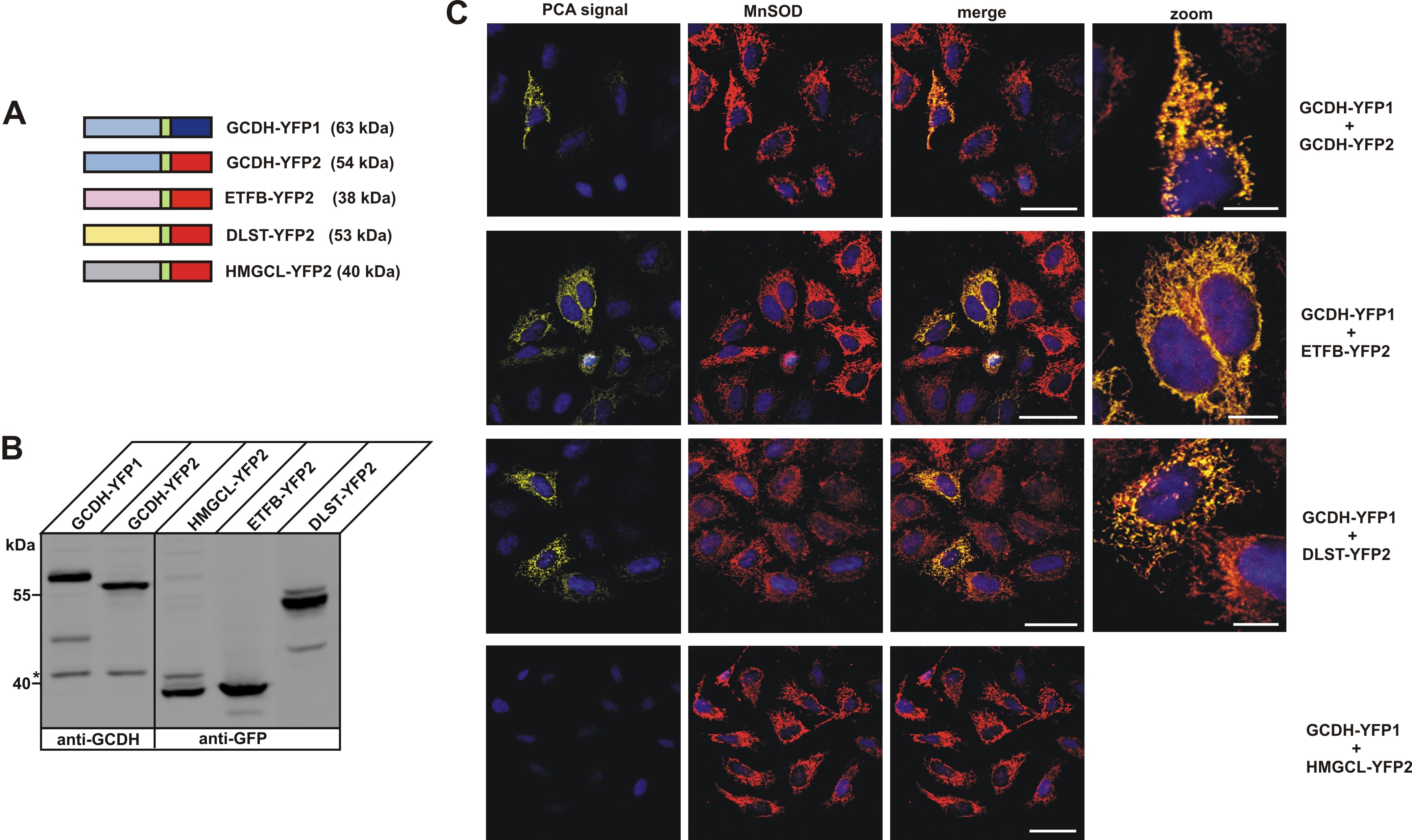

Supplement: Figure S4 — YFP fragment complementation assay and mitochondrial counterstaining. (A) Schematic composition of C-terminal YFP1 (dark blue) and YFP2 (red) fusion proteins of GCDH, ETFB, DLST, and HMGCL used in this study. The 10-amino acid linker (GGGGS)2 is indicated in green. The calculated molecular masses of the fusion proteins are shown in brackets. The mitochondrial matrix protein HMGCL was used as negative control. (B) Expression analysis in HeLa cells of all fusion proteins visualized by western blotting, using anti-GCDH and anti-GFP antibodies. *endogenous GCDH protein. (C) Fluorescence microscopy of the indicated co-expressed fusion proteins. Strong YFP fluorescence was observed in cells co-expressing GCDH-YFP1 with either GCDH-YFP2, ETFB-YFP2, or DLST-YFP2. No YFP fluorescence signal was observed when GCDH-YFP1 was co-expressed with HMGCL-YFP2. Nuclei were visualized using DAPI (blue). Mitochondria were counterstained with anti-MnSOD antibody. Merged signals indicate co-localization of PCA signal with MnSOD-positive mitochondria. Scale bars = 40 µm (merge) or 10 µm (zoom). (JPG) [file pone.0087715.s004.jpg]

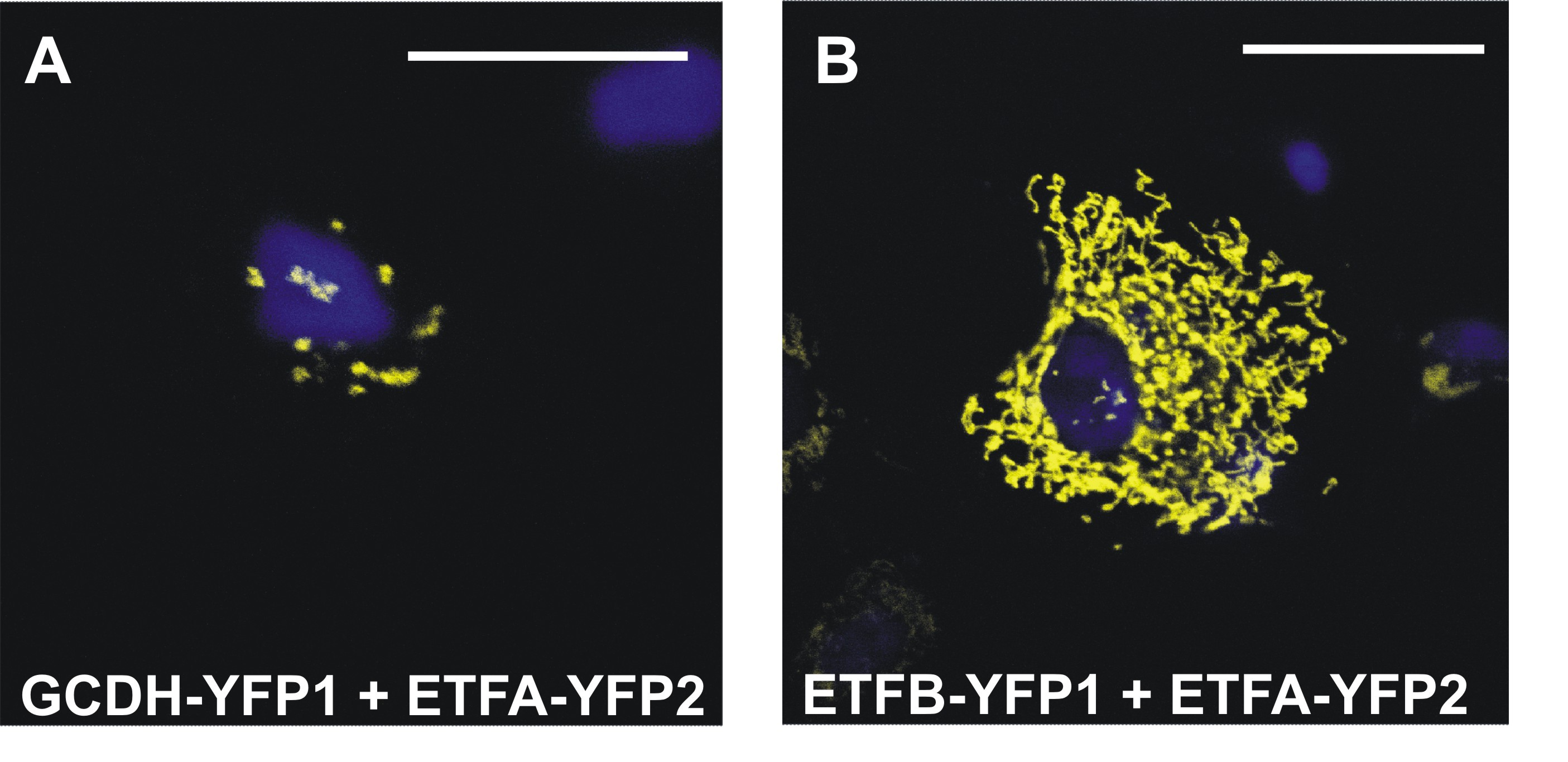

Supplement: Figure S5 — Interaction of GCDH with ETFA in vivo. (A) Fluorescence microscopy of fixed BHK cells co-expressing GCDH-YFP1 and ETFA-YFP2 fusion proteins showed a YFP fluorescence signal only in few mitochondria. (B) In contrast, co-expression of ETFB-YFP1 with ETFA-YFP2 revealed a strong fluorescence signal with a typical mitochondrial expression pattern. Nuclei were visualized using DAPI (blue). Scale bars = 40 µm. (JPG) [file pone.0087715.s005.jpg]

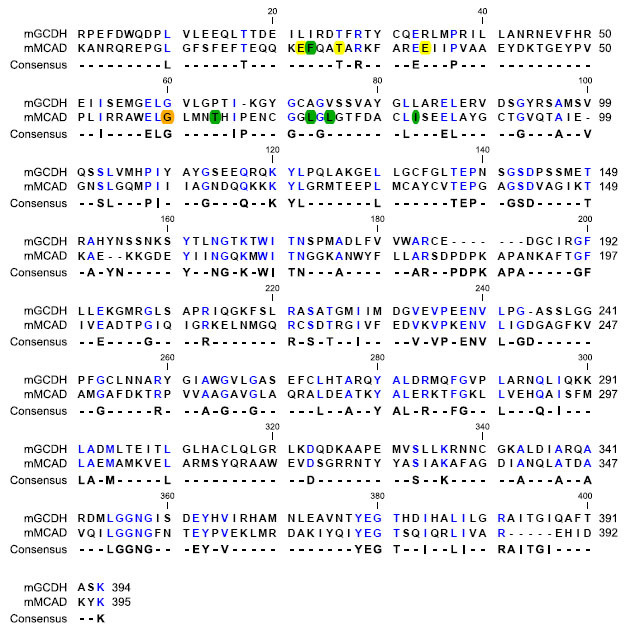

Supplement: Figure S6 — Comparison of the ETFB-binding site of MCAD with GCDH. Sequence alignment of mature GCDH and MCAD proteins. Identical amino acids are presented in blue. MCAD amino acid residues that have been reported to interact with ETFB [39] and to form hydrogen bonds (yellow), a hydrophobic pocket (green), or both (orange), are indicated. (JPG) [file pone.0087715.s006.jpg]
