# Supplementary material for: Interaction of Glutaric Aciduria Type 1-Related glutaryl-CoA Dehydrogenase with Mitochondrial Matrix Proteins
Source: PLoS One. 2014 Feb 3;9(2):e87715. doi: 10.1371/journal.pone.0087715 (PMC3912011; doi:10.1371/journal.pone.0087715)
Supplement: Table S1 — Sequences of primers used in this study. (DOC) [file pone.0087715.s007.doc]

**Supplementary Table S1: Sequences of primers used in this study**

| **Primer** | **Sequence** |
| --- | --- |
| TOPO-DLDfor | 5´-GACATGCAGAGCTGGAGTCGTGTG-3´ |
| TOPO-DLDrev | 5´-AAAGTTGATTGATTTGCCAAATG-3´ |
| TOPO-DLSTfor | 5´-GAGATGCTGTCCCGATCCCGCTGT-3´ |
| TOPO-DLSTrev | 5´-AAGCTTAAGATCCAGGAGGAGGACTCTG-3´ |
| TOPO-ETFAfor | 5´-CACCATGTTCCGAGCGGCGGCTCCG-´3 |
| TOPO-ETFArev | 5´-CCATTTTTTCTTCAATATCTCAGTC-3´ |
| TOPO-ETFBfor | 5´-CACCATGGCGGAGCTGCGCGTGCTC-´3 |
| TOPO-ETFBrev | 5´-AAGCTTAATCCGCCCAATCTCCTTCAGC-3´ |
| TOPO-HMGCLfor | 5´-CACCATGGCAGCAATGAGGAAGGCG-´3 |
| TOPO-HMGCLrev | 5´-GAGTTTACAGGTAGCCTGAGC-´3 |
| NdeI-ETFAfor | 5´-CATATGTTCCGAGCGGCGGCTCCGGGG-3´ |
| ETFA-HindIIIrev | 5´- AAGCTTTCATTTTTTCTTCAATATCTCAGT-3´ |
| NdeI-ETFBfor | 5´-CATATGGCGGAGCTGCGCGTGCTCGTAG-3´ |
| ETFB-HindIIIrev | 5´-AAGCTTAATCCGCCCAATCTCCTTCAGC-3´ |
| NcoI-mGCDHfor | 5´-CCATGGGCCGTCCCGAGTTTGACTGGCAG-3´ |
| GCDH-HindIIIrev | 5´-AAGCTTCTTGCTGGCCGTGAACGCCTG-3´ |
| YFP1u2for | 5´-TCTGCAGATATCGGTGGCGGTGGCTCTGGAGGT |
| YFP1-Arev | 5´- CGTTCTAGATTACTGCTTGTCGGCCATGATATA-3´ |
| YFP2-Arev | 5´-CGTTCTAGATTAGGATCCGTCGACCTTGTACAGCTCGTCCAT-3´ |
| YFP1-Brev | 5´-CGTCTCGAGTTACTGCTTGTCGGCCATGATATA-3´ |
| YFP2-Brev | 5´-CGTCTCGAGTTAGGATCCGTCGACCTTGTACAGCTCGTCCAT-3´ |

The restriction sites are indicated by underlined nucleotides.

The replaced codons corresponding to mutated amino acid residues are boldface.
