# Supplementary material for: Interaction of Glutaric Aciduria Type 1-Related glutaryl-CoA Dehydrogenase with Mitochondrial Matrix Proteins
Source: PLoS One. 2014 Feb 3;9(2):e87715. doi: 10.1371/journal.pone.0087715 (PMC3912011; doi:10.1371/journal.pone.0087715)
Supplement: Table S2 — LC-MS/MS analyses of the GCDH affinity chromatography elution fraction identifying mitochondrial and non-mitochondrial proteins. (DOC) [file pone.0087715.s008.doc]

**Supplementary Table S2: LC-MS/MS analyses of the GCDH affinity chromatography elution fraction identifying mitochondrial and non-mitochondrial proteins.**

| **gene** | **#of total peptides** | **score** | **peptide** |
| --- | --- | --- | --- |
| **ALDH2** | 4 | 41 | K.LGPALATGNVVVMK.V  K.EEIFGPVMQILK.F  R.TEQGPQIDETQFK.K  K.VSEQTPLTALYVANLIK.E |
| **DLST** | 2 | 96 | R.GLVVPVIR.N  R.NVETMNYADIER.T |
| **ETFB** | 1 | 20 | K.LSVVSVEDPPQR.V |
| **GLUD1** | 6 | 76 | K.YNLGLDLR.T  K.MVEGFFDR.G  R.TAAYVNAIEK.V  R.DDGSWEVIEGYR.A  K.IIAEGANGPTTPEADK.I  K.HGGTIPIVPTAEFQDR.I |
| **PRDX3** | 1 | 32 | R.GLFIIDPNGVIK.H |
| **ATP5A1** | 3 | 22 | K.NALGSSFIAAR.N  K.GIRPAINVGLSVSR.V  R.TGAIVDVPVGEELLGR.V |
| **ATP5B** | 3 | 72 | R.TIAMDGTEGLVR.G  R.IMNVIGEPIDER.G  R.AIAELGIYPAVDPLDSTSR.I |
| **CAT^1^** | 7 | 127 | K.LNILTAGPR.G  K.LNILTAGPR.G  K.DAQLFIQKK.A  K.NFSDVHPDYGAR.I  R.FSTVAGESGSADTVR.D  R.FNSANEDNVTQVR.T  R.AAQKPDILTTGSGNPIGDK.L |
| **DHRS4^2^** | 2 | 69 | K.NLAVELAPR.N  K.TNFSQVLWMDK.A |
| **UOX^1,3^** | 4 | 61 | K.DQFTTLPEVK.D  K.TTQSGFEGFIK.D  R.AQVYVEEVPWK.R  K.DYLHGDNSDVIPTDTIK.N |

1 = peroxisomal localization; 2 = localization unknown; 3 = no data available on human genome
